# Magnetically Driven Powerless Lighting Device with Kirigami Structured Magneto–Mechanoluminescence Composite

**DOI:** 10.1002/advs.202207722

**Published:** 2023-04-19

**Authors:** Michael Abraham Listyawan, Hyunseok Song, Ji Yun Jung, Joonchul Shin, Geon‐Tae Hwang, Hyun‐Cheol Song, Jungho Ryu

**Affiliations:** ^1^ School of Materials Science & Engineering Yeungnam University Gyeongsan 38541 Republic of Korea; ^2^ Electronic Materials Research Center Korea Institute of Science and Technology (KIST) Seoul 02792 Republic of Korea; ^3^ Department of Materials Science & Engineering Pukyong National University Busan 42601 Republic of Korea; ^4^ School of Advanced Materials Science and Engineering Sungkyunkwan University (SKKU) Suwon 16419 Republic of Korea; ^5^ KIST‐SKKU Carbon‐Neutral Research Center Sungkyunkwan University (SKKU) Suwon 16419 Republic of Korea; ^6^ Institute of Materials Technology Yeungnam University Gyeongsan 38541 Republic of Korea

**Keywords:** kirigami, lighting devices, magnetically‐driven devices, mechanoluminescence composites, ZnS:Cu

## Abstract

The energy crisis and global shift toward sustainability drive the need for sustainable technologies that utilize often‐wasted forms of energy. A multipurpose lighting device with a simplistic design that does not need electricity sources or conversions can be one such futuristic device. This study investigates the novel concept of a powerless lighting device driven by stray magnetic fields induced by power infrastructure for obstruction warning light systems. The device consists of mechanoluminescence (ML) composites of a Kirigami‐shaped polydimethylsiloxane (PDMS) elastomer, ZnS:Cu particles, and a magneto–mechano‐vibration (MMV) cantilever beam. Finite element analysis and luminescence characterization of the Kirigami structured ML composites are discussed, including the stress–strain distribution map and comparisons between different Kirigami structures based on stretchability and ML characteristic trade‐offs. By coupling a Kirigami‐structured ML material and an MMV cantilever structure, a device that can generate visible light as luminescence from a magnetic field can be created. Significant factors that contribute to luminescence generation and intensity are identified and optimized. Furthermore, the feasibility of the device is demonstrated by placing it in a practical environment. This further proves the functionality of the device in harvesting weak magnetic fields into luminescence or light, without complicated electrical energy conversion steps.

## Graphical abstract

A concept of magnetically driven powerless lighting device is introduced by combining mechanoluminescence material and magneto–mechano vibration of cantilever structure. Such device has the potential to be a sustainable and efficient alternative lighting source as it can be driven by stray magnetic fields. The design, optimization, and testing in practical environments are summarized and presented.

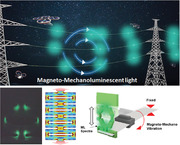

## 1 Introduction

**Figure 1:**
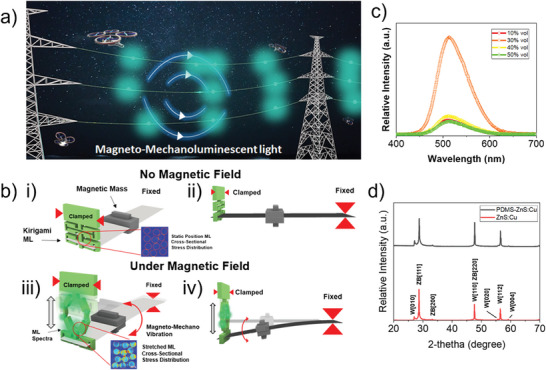
a) Illustration of the obstacle warning light systems when attached to transmission power lines for improving visibility and reducing the risk of flying object strikes. b) Conceptual schematic drawings of a magnetically driven powerless lighting device. The device is composed of two components: Kirigami structured PDMS‐ZnS:Cu mechanoluminescence (ML) composite and a MMV cantilever. b‐i) The static mode of the device when no magnetic field is applied to it and there is no stress distribution on the ML powder, b‐ii) the side perspective showing no deflection in the condition. The operation of the device under an applied magnetic field; b‐iii,iv) the cantilever structure vibrates due to magnetic torque, consequently exerting luminescence from the edges of the Kirigami structured ML section. The ML powder is acted upon by stress, as shown in the cross‐sectional illustration. In addition, ML composite characterization showcases c) the optimal composition for the optimal luminescence intensity and d) X‐ray diffraction of the composite and powder.

With recent technological advancements and the trend toward more electricity usage, a byproduct known as parasitic magnetic noise that is generated by most electrical infrastructures, such as transmission power lines, as well as everyday appliances, is now pervasive.^[^ ^1^, ^2^, ^3^ ^]^ Technologies that can convert and harvest this energy for useful work will be intriguing. For instance, lighting devices driven by stray magnetic fields have the potential to improve the automated inspection of power transmission lines^[^ ^4^ ^]^ and reduce the risk of collision for birds or inspection drones,^[^ ^5^ ^]^ as shown in **Figure** **1**a. In addition, the device does not require additional energy conversion steps such as rectification, regulation or electricity to light, which makes it a sustainable green technology.

Several devices with similar concepts, such as magneto–mechano–electric (MME) generators,^[^ ^6^, ^7^, ^8^ ^]^ which can convert magnetic fields to electrical energy and power wireless internet of things (IoT) sensors or other low‐power consumption electric devices, have been developed.^[^ ^9^, ^10^, ^11^ ^]^ An MME generator typically converts the magnetic field into a form of mechanical energy, utilizing a phenomenon called magneto–mechano vibration (MMV) from a cantilever structure.^[^ ^12^, ^13^, ^14^ ^]^ By employing similar concepts of magnetic‐to‐mechanical stimuli conversion, a powerless lighting device that utilizes a combination of mechanoluminescence (ML) material and MMV generated by ubiquitous stray magnetic noise from electric infrastructures can be designed. Note that a LED matrix is widely deployed in electrical towers, telecommunication towers, and skyscrapers to eliminate the risk for aviation.^[^ ^15^ ^]^ These LED‐based obstacle lighting systems for aircraft safety can be replaced with powerless and semi‐permanent illumination through a combination of ML and MMV under specific conditions where magnetic fields exist, such as around power transmission lines and electric towers in the future.

The fundamentals of ML materials have been extensively investigated,^[^ ^16^, ^17^ ^]^ and the application field is diverse, ranging from macro applications such as displays,^[^ ^18^ ^]^ elastic illuminating fabrics,^[^ ^19^ ^]^ strain sensors,^[^ ^20^ ^]^ and biomedical applications.^[^ ^21^ ^]^ Most of these application studies used an established ML material, that is, polydimethylsiloxane (PDMS)–ZnS:Cu, which^[^ ^22^ ^]^ exhibits high visible luminescence intensity^[^ ^23^, ^24^ ^]^ as well as elastic and recoverable luminescence for numerous applications. Despite these superior characteristics, ML design and shapes are essential for the device to produce luminescence because non‐contact excitation usually exerts only weak mechanical stimuli. For instance, the wind‐driven display requires the ML material to be shaped into smaller fabric‐like tube shapes to ease the excitation via wind compared to regular rectangular shapes.^[^ ^18^ ^]^ Thus, the structuring of ML materials into certain shapes can enable efficient luminescence emission even under weak mechanical stimuli, which will directly impact the function of the device to emit luminescence.

As the shape effect is critical, this study explores the effect of a unique shape, the “Kirigami” structure, which can improve functionality, stretchability, reconfigurability, and many other unique characteristics^[^ ^25^, ^26^, ^27^ ^]^ for various applications^[^ ^28^, ^29^, ^30^ ^]^ The Kirigami structure has the unique capability to transform flat sheets into a 3D structure, and there have been various breakthroughs in wearable sensors and devices that utilize this structure.^[^ ^31^ ^]^ The structure also significantly improves overall stretchability^[^ ^32^, ^33^ ^]^ Intrinsic stretchable geometry also amplifies the locally concentrated stress in specific parts.^[^ ^34^, ^35^, ^36^ ^]^ A similar structure was thoroughly studied for a similar elastic material, which is polyethylene terephthalate (PET), by Bartlett et al.^[^ ^37^ ^]^ The results showed that the structure significantly reduced stiffness while increasing the ultimate strain and the elastic range. The study also showed that the structure could sustain the rapid mechanical response. Thus, this structure is suitable to be implemented on PDMS–ZnS:Cu as a powerless lighting device that is subjected to rapid mechanical stimuli induced by stray AC magnetic noise from electric infrastructure.

This study introduces the novel concept of a magnetically driven powerless lighting device that combines an MMV from a cantilever structure with a Kirigami‐shaped ML composite. A cantilever MMV similar to the MME generator concept is created by applying an AC magnetic field to the cantilever structure with a magnetic mass. The lighting device (see Figure 1b) can be powered without any electricity, bypassing the usual energy conversion steps such as rectification and regulation of electricity in the energy harvesting‐based lighting device, highlighting its better efficiency. To enable efficient luminescence emission under MMV deflection, the incorporation of Kirigami structure is imperative as it reduces the rigidity significantly and creates effective stress concentrations inside the ML composite. Different Kirigami shapes and material dimensions are also characterized and optimized to achieve the highest luminescence behavior. The geometry of the Kirigami structured ML composite is optimized through finite element analysis (FEA) with several boundary conditions. We also investigate several factors of the device that increase luminescence by tailoring the ML particle content in ML (see Figure 1c) and device structure, including variations in the distance between the cantilever and the clamper.

## 2 Results and Discussion

### 2.1 Design and Optimization of Kirigami Structure With FEA

**Figure 2:**
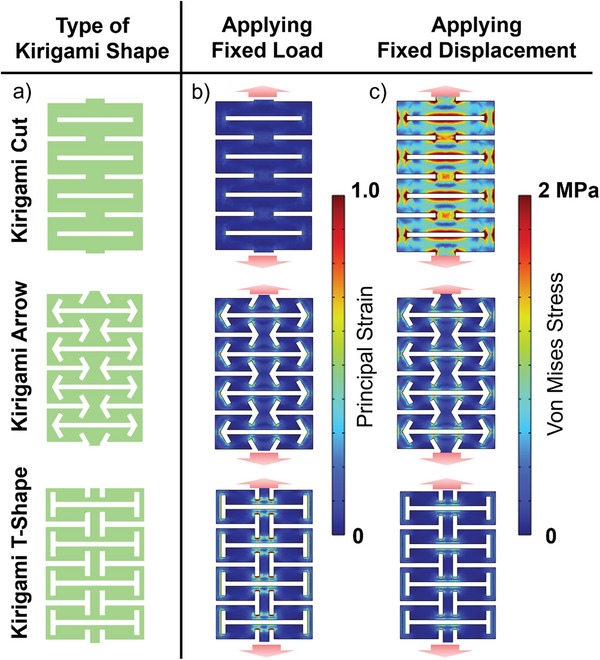
Finite element analysis (FEA) results of different Kirigami structures. a) Three types of Kirigami geometries: Kirigami cut, Kirigami arrow, and Kirigami T‐shape. The modeling is then applied with two different scenarios of fixed load (total force: 0.7 N) and fixed displacement perpendicular to the cuts (150 mm) boundary conditions. b) In the fixed load scenario, the principal strain distribution map is displayed, with a scale of 0–1.0. c) The fixed displacement scenario shows a stress distribution map with a maximum scale of 2 MPa. The red arrows indicate the displacement and load direction.

Incorporation of the ML composite into the Kirigami structure was simulated beforehand to understand the distribution of stress and strain, and the differences between each Kirigami structure that we designed, as shown in **Figure** **2**. The three Kirigami designs modeled for the simulations are called the cut shape, T‐shape, and arrow shape, as shown in Figure 2a. The motif of all the designs is based on the Kirigami‐engineered elasticity^[^ ^30^ ^]^ approach that utilizes only the cutting of the part. However, the incorporated design did not utilize the cutting method. Rather, we removed some parts of ML composite that mimicked the shape as the Kirigami‐engineered elasticity approach. The cut shape is a regular Kirigami‐engineered elasticity approach shape, which is widely used in other materials and applications.^[^ ^38^, ^39^, ^40^ ^]^ The T‐shape is a modified Kirigami structure that has additional inside cuts perpendicular to the main cuts; thus, creating the “T” shape; there are different names for this shape, such as the hybrid Kirigami shape;^[^ ^37^ ^]^ this shape significantly improves stretchability or strain. It is also a non‐protruding or minimalized buckling Kirigami structure owing to the additional cuts that rotate when the Kirigami structure is subjected to a uniaxial tensile load. Last, the arrow shape was chosen as we added an angle to the additional cut in the T‐shape to further analyze the effect of different cuts applied on the inside cuts of the Kirigami structure. In the FEA simulation, two different scenarios were applied to each Kirigami structure, namely, fixed displacement and fixed‐load conditions.

In Figure 2b,c, results show that the stress distribution was mainly concentrated at the edges of the cuts, similar to other Kirigami geometries.^[^ ^41^, ^42^, ^43^, ^44^ ^]^ Under the fixed displacement condition, a fixed stretching displacement of 150 mm was applied to all models and the strain distribution maps of the Kirigami structures were compared. The T and arrow shapes have a smaller area under stress; however, the stress distribution is much more localized, creating stress concentration areas on the edges, whereas the cut shape stress concentration area is wider and more distributed. This indicates that the T and arrow shapes have enhanced stretchability compared with that of the cut shape; however, the cut shape can exert higher stress with less displacement due to its higher rigidity. Meanwhile, for the fixed load condition, the load applied was ≈0.4 N (total force) along the *z*‐axis, which was perpendicular to the straight cut. Results show an abysmal stress distribution for the cut shape, whereas the Kirigami T‐shape and Kirigami arrow‐shape results display similar or even higher stress. This shows that the T and arrow shapes have better sensitivity to the load and can exert the same amount of stress using a relatively low load, similar to the MMV condition. The enhanced stretchability and load sensitivity are owing to the nature of the Kirigami structure, which creates isolated stress concentration areas around its edges. This creation of a stress concentration area mimics the stress concentration mechanism, which can be similarly observed in material defects.^[^ ^35^ ^]^ The comparison of the stress distribution indicates that the cut shape will have the highest luminescence intensity because it has more area with concentrated stress. Consequently, more ML powder will be activated through the stress; thus, the T‐shape with the lowest area of stress concentration will result in the lowest ML intensity. The thickness of the sample also affects the stress distribution, as shown in Figure S1, Supporting Information, which indicates a proportional relationship between the thickness of the structure and stress concentration.

The stress concentration mechanism is more evident when the two models of the arrow and T‐shape are compared. Referring to the stress distribution in Figure 2c, it can be observed that the stress concentration is more localized and concentrated for the T‐shape than for the arrow shape. The stress concentration is formed because the edges of the additional cuts turn into hinges that bend when a load is applied. The higher stress concentration is caused by the orientation of the additional cuts, which are directly perpendicular to the Kirigami linear cut. In contrast, the arrow shape has an ≈45° angle. These ≈90° angles of the T‐shape form an abrupt change in the stress flow and turn the edges into a hinge, thereby creating a higher stress concentration on the edges. The dimension surrounding the additional cut shaping the “T” is also thinner; thus, adding to the factor of increased stress. Meanwhile, the arrow‐shaped angle creates a much smoother change in the stress flow, and with its angle, the edge cannot rotate as far as it can with the T‐shaped angle. In addition, this shape has a much greater thickness and dimension surrounding the inner cuts that shape the arrow.

The FEA results of this Kirigami model focus on the stress generated and principal strain that is created with respect to the uniaxial load applied. The study of the non‐protruding Kirigami structure^[^ ^33^ ^]^ for stretchable piezoelectric nanogenerators by Lee et al. also showcases the FEA analysis, with emphasis on the increase in strain as they applied the “T” Kirigami shape. Our results concentrate on the stress generation for this specific Kirigami model as it affects the ML exertion. Other studies, such as the programmable kiri‐kirigami shape^[^ ^29^ ^]^ by Yin et al. show only the stress concentration effect that occurs on its edges when it is applied with a similar load.

### 2.2 Characterization of Luminescence Behavior of ML Composite With Kirigami Structures

**Figure 3:**
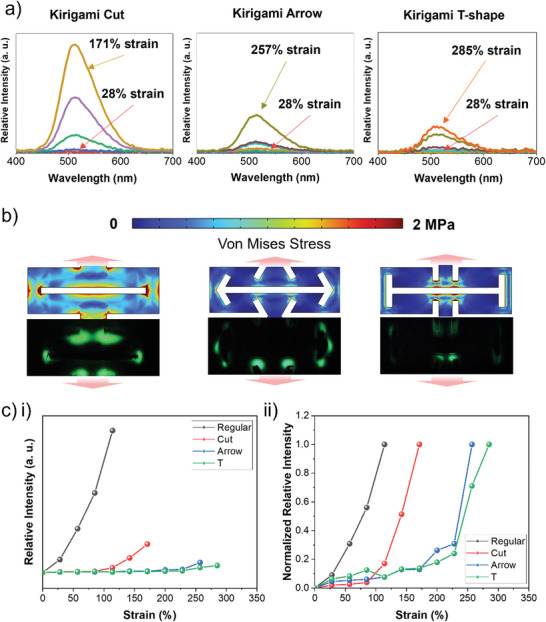
Characterization of ML intensity of Kirigami ML composites: a) Different ML Kirigami structures are applied with controlled strain, and the intensities are measured by the spectrometer. b) Figures of characterization are taken when the highest constant strains for cut shape, arrow shape, and T‐shape, that is, 171%, 257%, and 285%, respectively, are applied and compared with the stress distribution map taken from the FEA simulation; red arrows indicate the stretching direction. c‐i) The maximum intensity as a function of applied strain for the Kirigami structure is plotted for a direct comparison. c‐ii) The normalized relative ML intensity clearly shows where each Kirigami structure reaches its maximum ML intensity.

To further analyze the effect before incorporating ML composite into the design and fabrication of the magnetically driven powerless lighting device with the ML composite, luminescence characterization of the ML material is required. The detailed fabrication process of the ML composites is described in the Experimental Section. Characterization was conducted by applying an increasing strain up to the limit of each Kirigami structure. Results shown in **Figure** **3**a indicate that the cut and T‐shapes exert the highest and lowest luminescence intensities, respectively. The displacement ranges of various shapes are also different, which indicates that each shape has distinct stretchability and, consequently, rigidity. The strain relation indicates that the T‐shape has the widest range of strain, whereas the arrow shape has a higher maximum strain than the cut shape. This signifies that the additional inside cut improves the conventional Kirigami design, regardless of its angle. As the only control variable in the characterization test was the strain or displacement of the linear stage, the load could not be further analyzed; however, the relationship between load and ML intensity can be explicated through the fixed‐load applied simulation. This further confirms that the T and arrow shapes can generate high strain, and, consequently, high stress, even with a low tensile load applied. Therefore, the increase in the maximum strain in the test produces results similar to those of the stress simulation.

Furthermore, there are several reasons for the high intensity shown by the Kirigami cut shape. The stress distribution in the simulation indicates different stress distributions, and the cut shape shows a wider stress distribution than other shapes. In Figure 3b, we compare the snapshot obtained during the characterization test with the stress distribution of the FEA simulation results. The comparisons of ML phenomena between different Kirigami structures are also documented in Video S1, Supporting Information. The areas that exhibit luminescence match well with the simulated stress distribution map when compared directly. As the area is wider in the cut shape, more ZnS:Cu powders are homogenously distributed inside the PDMS matrix. Consequently, with a wider stress distribution area, there will be more ZnS:Cu powder activated in the cut shape compared to that in other shapes that show a more localized stress distribution. The T‐shape in this case has the smallest stress distribution area; and thus, shows the lowest luminescence intensity. In addition, the rigidity of the shape also affects the luminescence intensity. As the cut shape has higher rigidity, the maximum load that can be applied is relatively higher; therefore, it has a better intensity compared to that of the T‐ or arrow shapes, which have lower rigidity. This study finds that there is a stretchability trade‐off with the various Kirigami shapes as additional cuts or hybrid cuts are added.

As shown in Figure 3c, we compared the luminescence intensities of different Kirigami structures and the regular rectangular shape without a Kirigami shape. There is an evident relationship between stretchability and luminescence intensity, as shown in the first graph. The Kirigami T‐shape has the highest strain while exerting a significantly lower luminescence intensity, and the strain is tripled compared to that of the regular shape. Although the Kirigami T‐shape has the lowest intensity, the high strain indicates an increase in stretchability and, consequently, lower rigidity.

The increase in strain is also significantly higher compared to that in another Kirigami shape study with protruding mechanisms.^[^ ^45^ ^]^ This comparison confirms that the Kirigami designs are a much better fit for high flexibility and high strain applications such as MMV. Therefore, this shape is highly sensitive to load and can be used in a wider range of applications. Results of similar studies that employ identical Kirigami shape^[^ ^37^ ^]^ by Bartlett et al. show similar findings of the decrease in stiffness as hybrid cuts are applied. However, the previous study only conducted the experiment on PDMS and did not examine its effect on stress concentration or mechanoluminescence application. Our findings show the normalized intensity graph indicating a unique mechanism in which the intensity suddenly increases at a certain strain. The data emphasizes the relation between various Kirigami cuts and mechanoluminescence for potential application in ML based lighting and other devices. This shows that the ML spectra have a certain range of stress to activate optimally, and a clear trend can be observed from the comparison of different shapes.

### 2.3 Design and Optimization of Magneto–Mechanoluminescence (MML) Device

**Figure 4:**
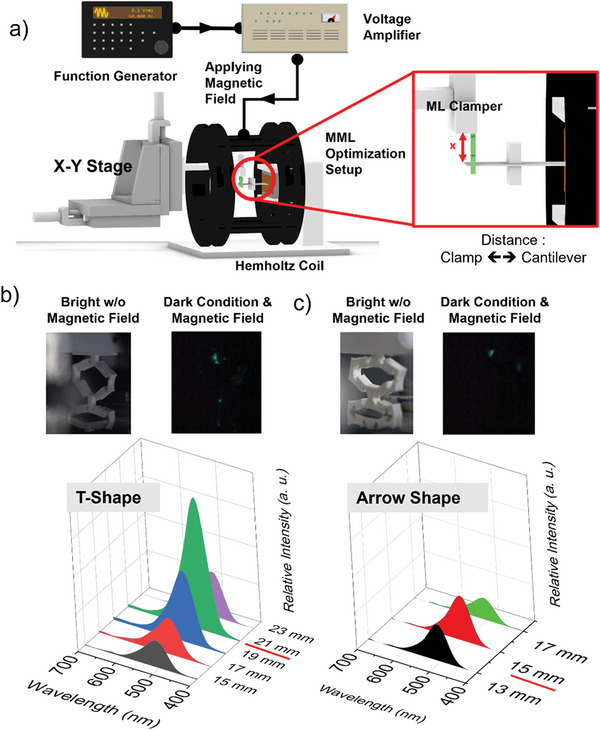
Optimization of the magnetically driven powerless lighting device (MML): the setup utilizes a Helmholtz coil to provide an AC magnetic field that will induce the cantilever with magneto–mechano vibration (MMV). As can be seen in schematic a) the device is placed inside the Helmholtz coil, while for the ML clamper, the *X–Y* stage is used to adjust the distance between the clamp and cantilever for optimization. The results can be seen for two Kirigami structures: the b) T‐shape and c) arrow shape through photograph and intensity versus wavelength plot. Graphs show several intensities at different clamper positions.

The magneto–mechanoluminescence (MML) concept for a magnetically driven powerless lighting device constitutes a titanium‐based MMV cantilever structure with a thickness of 0.35 mm attached to the magnet mass with a weight of 17.6 g and ML Kirigami composite at the tip. This combination requires several adjustments to two variables, namely, the distance between the cantilever and the ML Kirigami clamper and the different Kirigami structures because each shape has different elastic and ML characteristics. These variables are important for optimization because they directly and significantly affect the luminescence intensity of this device; and hence, its overall performance. The full experimental setup is shown in **Figure** **4**a. We maintained the AC magnetic field generated using the Helmholtz coil constant and uniform at ≈10 Oe and at a frequency of 60 Hz. The cantilever was attached to a magnetic mass to create flexural deflection when an AC magnetic field was applied. As mentioned above, we call this deflection an MMV because it converts magnetic energy into mechanical vibration energy. This concept has been further discussed and studied in magneto–mechano–electric (MME) generators.^[^ ^14^ ^]^ The relationship between the MMV amplitude and environmental conditions or device design parameters such as magnetic field, magnet mass, cantilever, and load generated has been thoroughly studied by Kang et al.^[^ ^46^, ^47^ ^]^ Similar to the MMV on this device, the force due to the permanent magnet mass was affected by the dimensions of the magnet, the remnant flux of the magnet, the magnetic field applied, and the shape of the beam. The optimization used a cantilever structure with a length of 60 mm. This rather long profile, compared to other cantilever structure devices, affects the magnitude of deflection. As this device relies solely on flexural deflection as a mechanical stimulus, the deflection must be sufficiently high to stretch ML Kirigami. This also correlates with the luminescence intensity measurement as a high deflection will generate a higher intensity. The flexural deflection recorded in Figure S2, Supporting Information shows a significant reduction of ≈4 mm when the ML Kirigami is placed at the tip of the cantilever. The flexural deflection is ≈10 mm without any ML Kirigami part attached to the tip of the cantilever. The 4 mm decrease in deflection indicates that ML Kirigami has a dampening effect on the MMV deflection but much less than regular plate‐type ML.

First, we analyzed the effect of the distance between the placement of the Kirigami ML clamper and the cantilever structure on the luminescence intensity. The designed cantilever structure with the magnetic mass achieved a 60 Hz resonance frequency. This frequency was chosen so that the device could be adapted into a practical environment directly because most electrical appliances run on 60 Hz AC electricity. In Figure 4b,c, we can see that an optimal distance is required to acquire optimal luminescence intensity. Two Kirigami structures were tested, namely, an arrow shape and a T‐shape. Video S2, Supporting Information, shows the luminescence exerted during optimization. These shapes were chosen because the cut shape rigidity is too high and requires a much higher load to deform the Kirigami ML and exert luminescence. In this case, the load of magnetic torque deflection is insufficient to apply any displacement to the cut shaped Kirigami ML. Therefore, the cut shape was omitted from this device optimization because it completely dampens the MMV deflection. As for the other Kirigami shapes, the load required to deform the Kirigami ML is less, as shown by Bartlett et al. and also in the FEA results with fixed load scenarios. Therefore, these shapes will be a better fit for the design of our lighting device, which solely depends on the MMV deflection for the mechanical stimuli. In comparison, the T‐shape exhibits a higher luminescence intensity than the arrow shape. The Kirigami T‐shape also possesses a wider range of distance between the ML clamper and cantilever, that is, 15–23 mm, whereas the Kirigami arrow shape shows an overall lower intensity with a luminescence range of 13–17 mm. Further decreasing or increasing the range might lower the luminescence to a level that the spectrometer cannot detect, and the luminescence cannot be visibly observed.

The results show that the maximum and optimal distances for each shape also differ. The Kirigami T‐shape shows maximum intensity at a distance of 21 mm, whereas the arrow shape shows maximum intensity at a distance of 15 mm. Both shapes are similar in that if we increase the distance between the ML clamper and cantilever over the optimal distance, the luminescence intensity drops and vice versa. This is because ML Kirigami affects the MMV as a dampening factor. As shown in Figure S2, Supporting Information, the deflection is reduced when compared with and without ML Kirigami. According to Hooke's law, displacement only works linearly until a certain threshold is reached. The same principles can be applied here; therefore, as we increase the distance, the same load acting on ML Kirigami cannot create the same amount of displacement; thus, dampening the deflection, which also occurs if we excessively decrease the distance. However, results show that the intensity of the T‐shape is higher than that of the arrow shape, whereas in Kirigami ML characterization, the arrow shape has a brighter intensity. It indicates that the arrow shape is much more rigid and dampens the MMV slightly more than the T‐shape. Nevertheless, a consistent trend between characterization and FEA results shows that the T‐shape generates a higher maximum stress compared to that of the arrow shape when a fixed load is applied; thus, indicating that the ML mechanism is activated by stress and its intensity is influenced by the magnitude of the stress. Thus, the T‐shape is the most suitable Kirigami structure to be incorporated into this device, and there is an optimal distance between the ML clamper and cantilever. In addition, we tested the effect of magnetic field magnitude on device intensity using the T‐shape Kirigami with optimal settings. Figure S3, Supporting Information, clearly shows a proportional relationship as intensity increases with increasing magnetic field. The intensity can be observed starting from 10 Oe based on the dimensional design of the device.

### 2.4 Device Applicability in a Practical Environment

**Figure 5:**
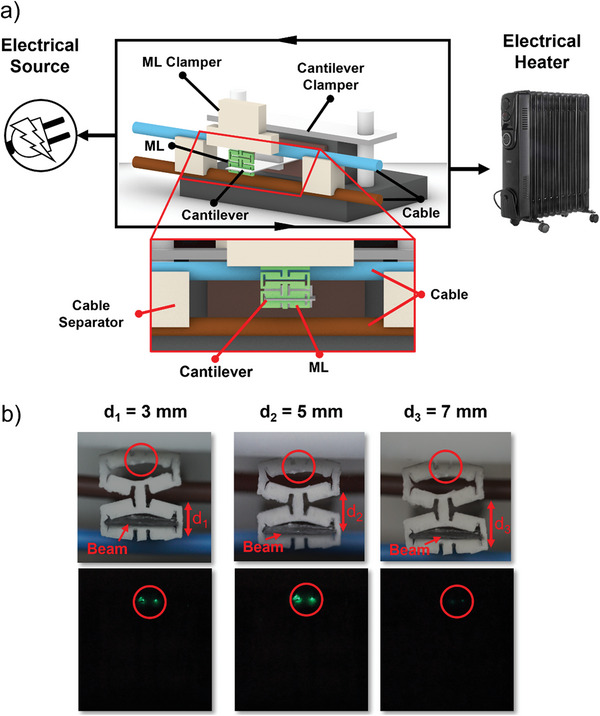
Operation of a magnetically driven powerless lighting device (MML) under practical conditions. The AC magnetic field is induced by the electrical heating appliance current. The device is placed between the two cables with different poles. In schematic a) custom 3D‐printed cable separators are used to adjust the distance of the cable. b) The photos show the luminescence exerted under practical conditions with variations in the gap dimensions between cables. The video taken during the operation is provided in Video S3, Supporting Information.

To demonstrate the applicability of this device in a practical situation, the Helmholtz coil was removed and the MML device was placed between the power cables of the electric appliance. The coil was removed as it provides ideal magnetic field conditions not found in practical settings, such as electric power cable infrastructures. An electrical portable heater (2.5 kW) was used as the current load, with a flowing current of ≈11 A at 220 V, which generated a magnetic field of ≈4.4 Oe. The size of the cantilever was scaled to further reduce the magnetic mass of the cantilever. Consequently, the Kirigami ML was scaled down to reduce the damping effect of the cantilever deflection. The distance (*d*_i_) of the cable along one vertical axis was also varied using a customized cable separator, as shown in **Figure** **5**. As shown in Video S3, Supporting Information on the application part, the distance (*d*_3_) shows dimmer luminescence compared to the other distances (*d*_1_ and *d*_2_). This distance effect was consistent with the basic theory that the magnetic field decreases with increasing distance from the cable. Despite the differing luminescence intensities, the device remains functional and can be driven solely by the very weak stray magnetic noise from electrical appliances.

## 3 Conclusion

The combination of the titanium cantilever structure MMV and ML composite with the Kirigami structure enables the operation of a magnetically driven powerless lighting device based on the MML principle. The Kirigami structure in the ML composite significantly reduces the rigidity and improves the stretchability. This unique change in characteristics enables the ML composite to exert stress at several localized points, owing to the stress‐concentrating nature of the Kirigami structure. The concentration points created were highly dependent on the shape of the Kirigami structure. Although Kirigami‐structured ML composites have less intense luminescence characteristics than unshaped ML composites, the incorporation of ML into Kirigami broadens the utilization prospects of ML for other applications. As a relatively low load can activate luminescence, the flexural deflection caused by the magnetic torque is sufficient to apply displacement to the Kirigami‐structured ML composite. The MML device is then further optimized for a functional application through variables such as the material of the cantilever, the distance of the ML clamper, the cantilever structure, and a comparison between different Kirigami ML shapes. The results of the optimization show that the device still has limitations, especially in the intensity that it produces. It is rather dim at its current level could not be utilized for practical lighting. However, additional modifications could improve its functionality, such as increasing the magnetic field input by using a magnetic flux concentrator or creating modular designs that improve the combined intensity and allow for its application as a powerless lighting device. Overall, this current device can generate visible luminescence signifying its potential for functionality, and operates in a 60 Hz AC stray magnetic noise without any electric power conversion steps.

## 4 Experimental Section

### FEA Modeling

3D FEA modeling was conducted using the COMSOL 6.0 Multiphysics software (COMSOL, Sweden). Different Kirigami shapes were defined beforehand using other CAD modeling software (Autodesk Inventor, Autodesk) for simpler geometry creation. It was then imported into COMSOL for further analysis of stress and strain distribution with different external stimuli applied. Based on the design presented in Figure 2a, two different boundary conditions were applied for each geometry. The differing factor in these scenarios was the external stimuli: the first scenario applied a fixed displacement condition (Figure 2b), whereas in the second scenario, a fixed load condition was applied (Figure 2c) to the different Kirigami structure models. The mechanical properties of the material were acquired through a tensile test, such as Young's modulus (≈2.2 MPa), as shown in Figure S4, Supporting Information. The density was calculated using the volumetric percentage combination of the elastomer composite (1.906 kg m^−3^), and Poisson's ratio was assumed to be 0.5, which is equal to PDMS. These properties were input as user‐defined material parameters in COMSOL. The mesh was defined through a software‐automated mesh generator, and the simulation was performed under steady‐state conditions and a time‐dependent study to acquire the stress distribution as the load was applied to the structure (see Video S4, Supporting Information). An additional study of the stress distribution was also performed with the variation in sample thickness, as shown in Figure S1, Supporting Information.

### Kirigami ML Materials Fabrication

The PDMS elastomer and curing agent (Sylgard 184, DOW Chemicals, USA) were mixed in a ratio of 10:1. ZnS:Cu powders (average particle size of 15 µm; LP‐6845, Lonco Company Limited, Hong Kong) as ML material was added later into the mixture to ensure the correct volumetric ratio of 30%, as shown in Figure 1c, because a 30% volumetric ratio exerts the highest luminescence.^[^ ^24^ ^]^ The mixture was then degassed for 30 min in a vacuum desiccator to remove any bubbles formed during the mixing. Subsequently, the degassed mixture was poured into a 3D‐printed, customized Kirigami mold made by a FORM3 series commercial 3D printer (Formlabs, USA). The mixture was cured in an oven at 70 °C for 2 h. Last, the mold was cooled, and the sample was removed from the mold. The holistic process for fabricating Kirigami ML materials is shown in Figure S5, Supporting Information. The solidified ML elastomer was then observed for the distribution of ZnS:Cu powder inside the PDMS matrix through cross‐sectional SEM observation, as shown in Figure S6, Supporting Information. It was confirmed that this method results in a homogeneous distribution similar to that of the previous study by Listyawan et al.^[^ ^24^ ^]^ The composite was also characterized using X‐ray diffraction and photoluminescence spectra, which are shown in Figure 1 c,d; Figure S7, Supporting Information.

### Characterization of Luminescence Behavior of ML Composite With Kirigami Structure

A customized linear stage (X‐LSM‐E, Zaber, Germany) and a spectrometer (BLUE Wave Spectrometer, Stellar Net Inc., USA) were used to measure the luminescence intensity of the ML composites with a Kirigami structure. Customized clampers were added to the linear stage for the sample, and the sample was stretched on one axis for multiple cycles. Python‐based programming was used to control the linear stage to provide multiple reciprocal single‐axis movements at a relatively high speed with a maximum of 104 mm s^−1^. Both the linear stage and spectrometer were connected to a computer for simultaneous data acquisition and a stage controller station. Then, the spectrometer was attached to an extended probe that could be placed close to the sample to enable luminescence intensity measurement. The probe was also placed near the edges that exerted the highest intensity compared with that of other parts of the sample. The setup was then placed inside a dark box to further isolate any external light other than the ML exerted by the materials. The entire setup is schematically shown in Figure S8, Supporting Information.

### Characterization of MML Device

The experimental setup required several components, that is, the cantilever structure for MMV torque, the Helmholtz coil that could generate an AC magnetic field, and an external clamper for the ML material that would be coupled with the cantilever. The MMV cantilever beam was made of an elastic grade 5 titanium alloy plate (60 mm × 20 mm × 0.35 mm) and a NdFeB permanent magnet‐proof mass (17.6 g). The AC magnetic field was tuned using a function generator (WF1946A, NF Corporation, Japan) and bipolar amplifier (HSA4014, NF Corporation, Japan). The frequency could be tuned through the function generator to enable testing at different frequencies because different structures have different resonance frequencies. The clamper structure was an *XY*‐movable stage that could be adjusted to optimize the luminescence exerted by the MML design further. This setup was also placed inside an enclosed dark environment to reduce any other light noise that might affect the luminescence intensity data. Luminescence data were measured with a spectrometer for luminescence characterization that utilized the same fiber‐optic probe utilized in the Kirigami ML characterization.

### Operation of MML Device in a Practical Environment

For the operation of the MML device, a setup utilizing an electric heater with a rated current of ≈11 A that generates an AC magnetic field around the power cable was set up. Subsequently, two connections were placed near the magnet mass and separated using a customized 3D‐printed separator. The dimensions of the cantilever were 43 mm × 9 mm × 0.35 mm in length, width, and thickness, respectively. The NdFeB permanent magnet proof with a mass of 3 g was attached to the end of the opposite side of the clamp. As for the Kirigami ML, the shape that the authors employed was a T‐shaped Kirigami with dimensions of length, width, and thickness of 8 mm × 10 mm × 1 mm. The cantilever and Kirigami ML were clamped using a 3D‐printed clamper, and the distance was scaled to ≈10 mm, that is, half of the optimal distance. This setup was also placed in an isolated dark container to limit any external light exposure, and photos and videos were taken using a DSLR camera with a frame rate of 30 fps.

## Conflict of Interest

The authors declare no conflict of interest.

## Supporting information

Supporting Information

Supplemental Video 1

Supplemental Video 2

Supplemental Video 3

Supplemental Video 4

## Data Availability

The data that support the findings of this study are available from the corresponding author upon reasonable request.
