# Supplementary material for: Magnetically Driven Powerless Lighting Device with Kirigami Structured Magneto–Mechanoluminescence Composite
Source: Adv Sci (Weinh). 2023 Apr 19;10(17):2207722. doi: 10.1002/advs.202207722 (PMC10265097; doi:10.1002/advs.202207722)
Supplement: Supplementary file 1 — Supporting Information [file ADVS-10-2207722-s003.pdf]

ADVANCED SCIENCE
Open Access

Supporting Information

for *Adv. Sci.*, DOI 10.1002/advs.202207722

Magnetically Driven Powerless Lighting Device with Kirigami Structured Magneto–Mechanoluminescence Composite

Michael Abraham Listyawan, Hyunseok Song, Ji Yun Jung, Joonchul Shin, Geon-Tae Hwang, Hyun-Cheol Song and Jungho Ryu**

Supporting Information

Magnetically Driven Powerless Lighting Device with Kirigami Structured Magneto-Mechanoluminescence Composite

Michael Abraham Listyawan[1], Hyunseok Song[1], Ji Yun Jung[1], Joonchul Shin[2], Geon-Tae Hwang[3], Hyun-Cheol Song[2,4,5*], and Jungho Ryu[1,6*]

[1]School of Materials Science & Engineering, Yeungnam University, Gyeongsan 38541, Republic of Korea

[2]Electronic Materials Research Center, Korea Institute of Science and Technology (KIST), Seoul 02792, Republic of Korea

[3]Department of Materials Science & Engineering, Pukyong National University, Busan 42601, Republic of Korea

[4]School of Advanced Materials Science and Engineering, Sungkyunkwan University (SKKU), Suwon 16419, Republic of Korea

[5]KIST-SKKU Carbon-Neutral Research Center, Sungkyunkwan University (SKKU), Suwon 16419, Republic of Korea

[6]Institute of Materials Technology, Yeungnam University, Gyeongsan, 38541, Republic of Korea

*Corresponding Author: Jungho Ryu and Hyun-Cheol Song, E-mail: jhryu@yu.ac.kr, hcsong@kist.re.kr

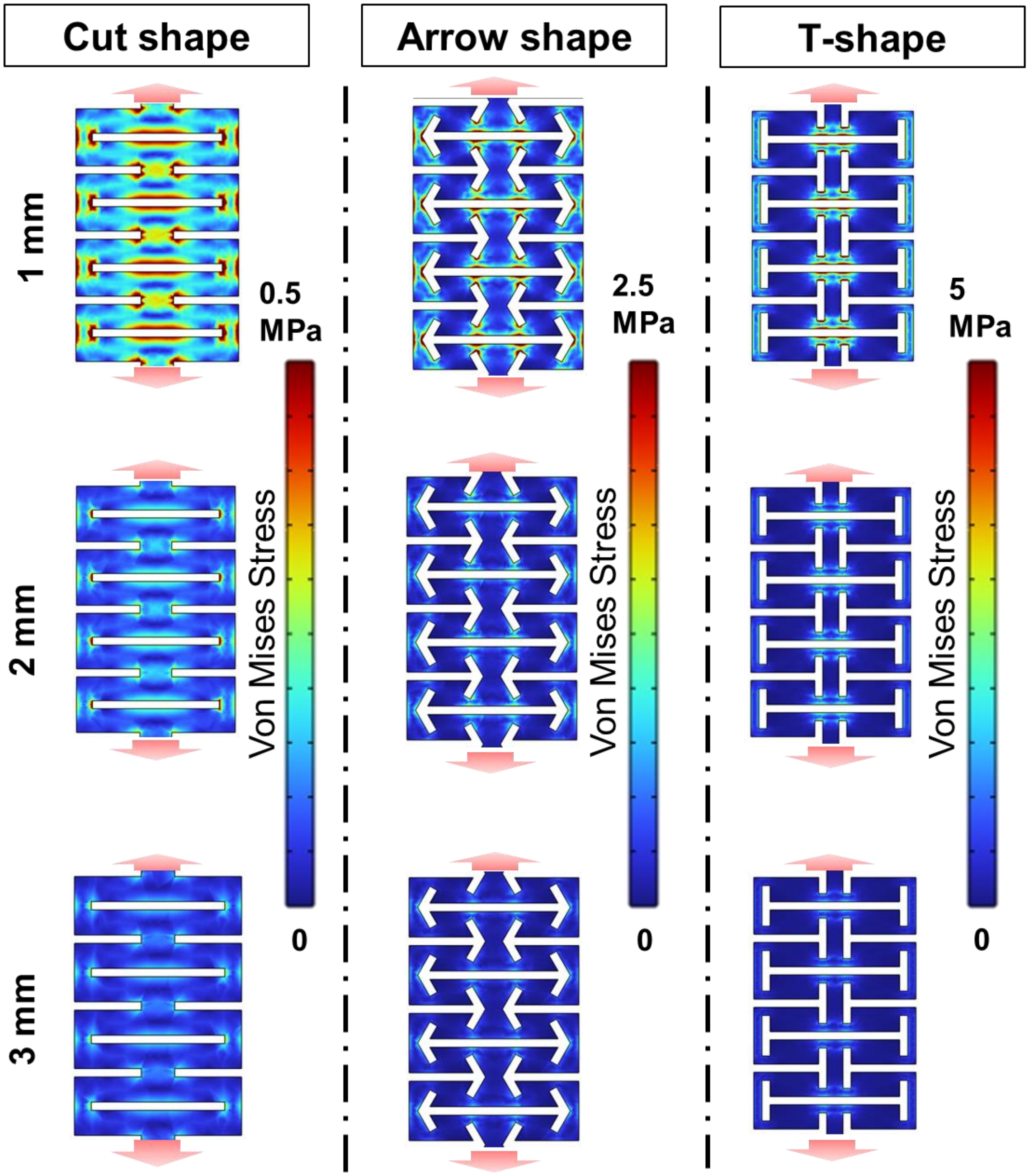

Figure S1 Finite Element Analysis of 3 Kirigami shapes (Cut shape, Arrow shape, and T-shape) and variating thickness starting from 1mm to 3mm. The reddish arrow indicates the direction of the load applied. The magnitude of the load is identical throughout all models and each model has a different stress scale. The Cut shape scale has a maximum of 0.5 MPa, Arrow shape has a higher value of 2.5 MPa, and the T-shape possesses the highest maximum value of 5 MPa.

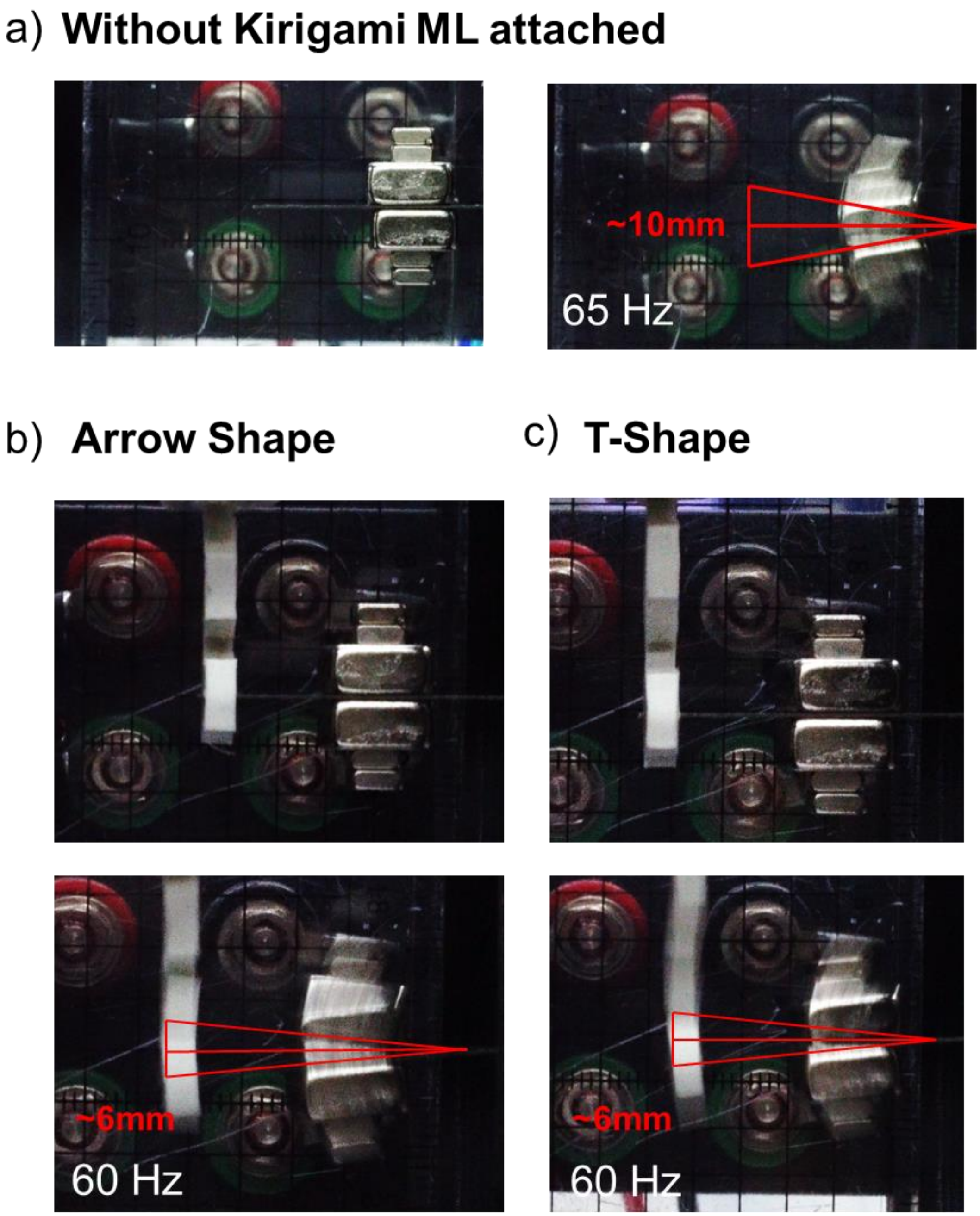

Figure S2 Deflection measurement is carried out through image measurement since the deflection is relatively high. The image taken also includes a ruler as a manual scale bar. The first measurement (a) is taken without any ML structure, it reaches a resonance frequency of 65 Hz. Then deflection measurements are taken for different Kirigami ML shapes attached which are arrow shape (b) and T-shape (c), with an approximate resonance frequency of 60 Hz.

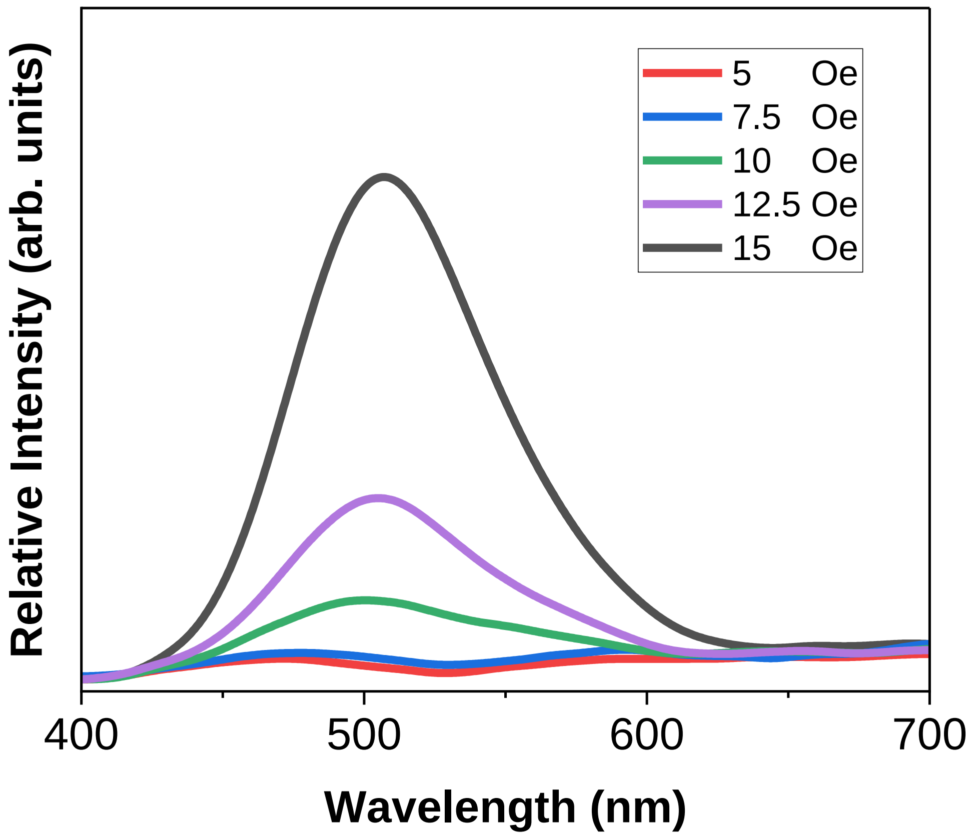

Figure S3 The effect of magnetic field on the lighting device intensity, the magnetic field magnitude starts from 5 Oe to 15 Oe. Device performance is visibly observed starting from 10 Oe and increasing as magnetic field is increased.

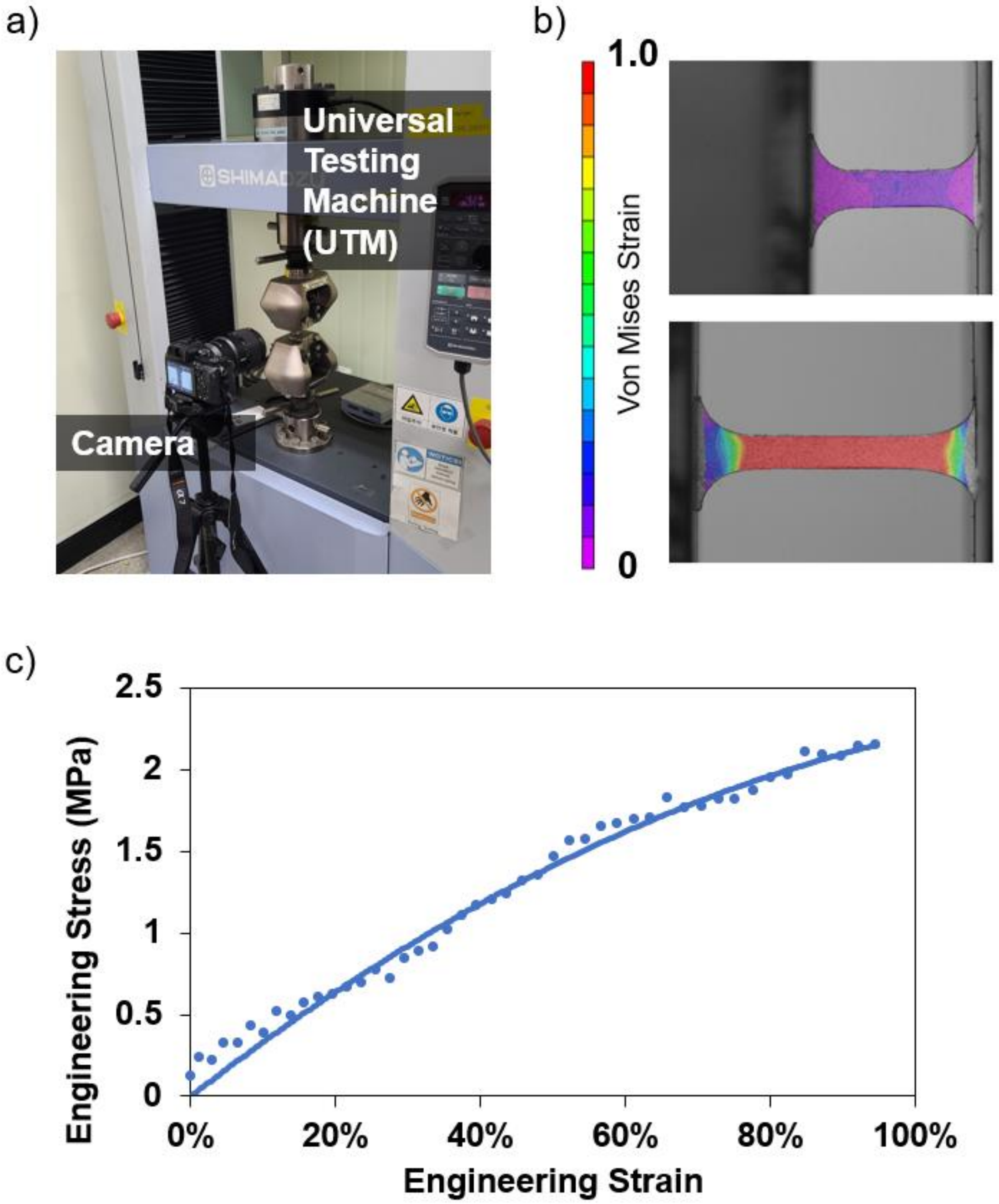

Figure S4 Tensile Test of PDMS-ZnS:Cu (a) the setup shown here utilizes a universal testing machine to precisely determine the load applied, and the video taken from the camera is used to measure strain by using digital image correlation (DIC). (b) The image processing of DIC determines the strain along the gage length. Lastly, the data combined from UTM and DIC (c) is able to plot a stress-strain graph that clearly shows mechanical characteristics.

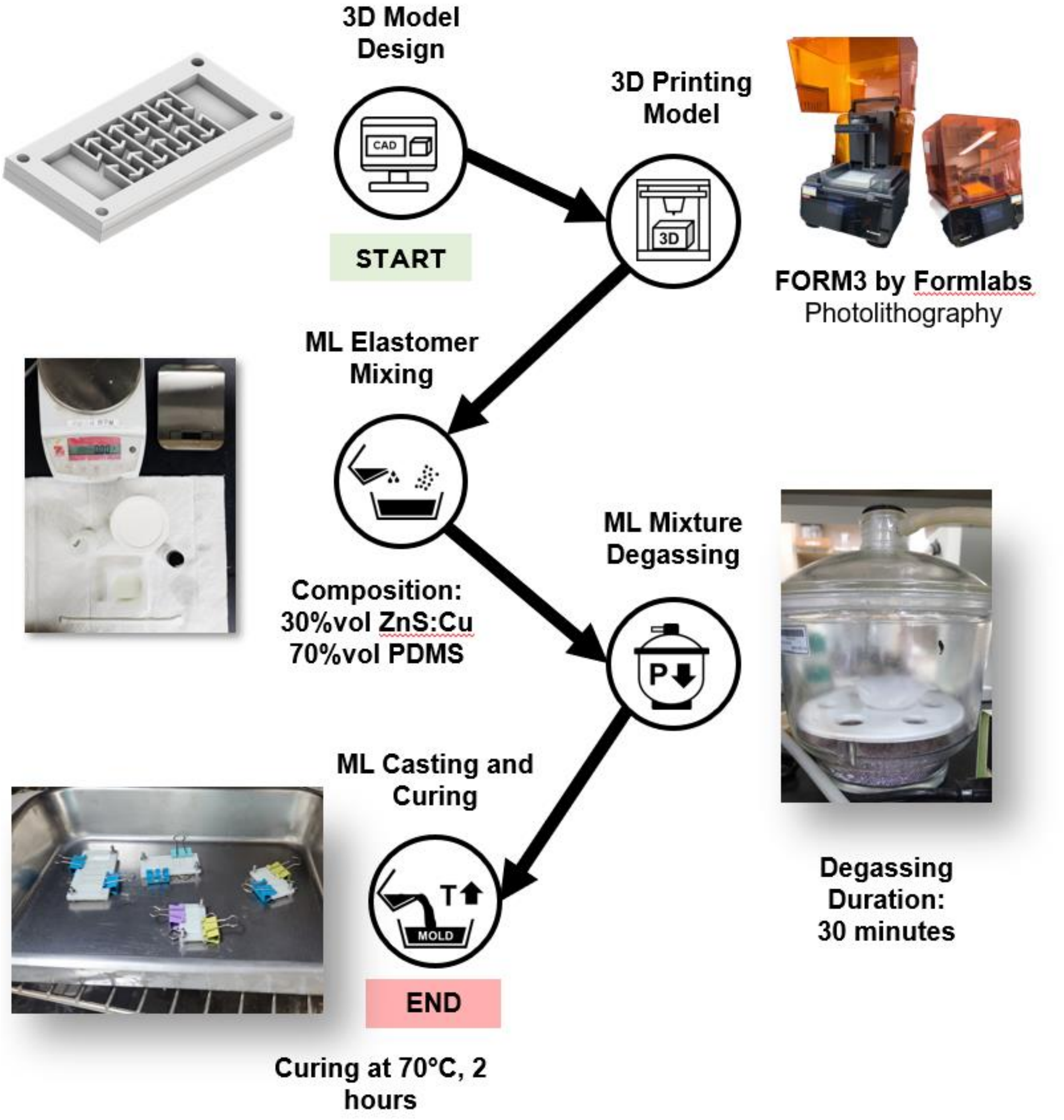

Figure S5 There are a few methods employed for fabricating the PDMS-ZnS:Cu ML elastomer into intricate Kirigami shapes. It is comprised of 5 steps that include modeling specific molds made by a 3D printer. Then, the mixed elastomer is filled into the mold. The mixture composition has been optimized for 30% volumetric content of ZnS:Cu. After degassing, the ML mixture is poured into the mold and cured at 70°C for 2 hours

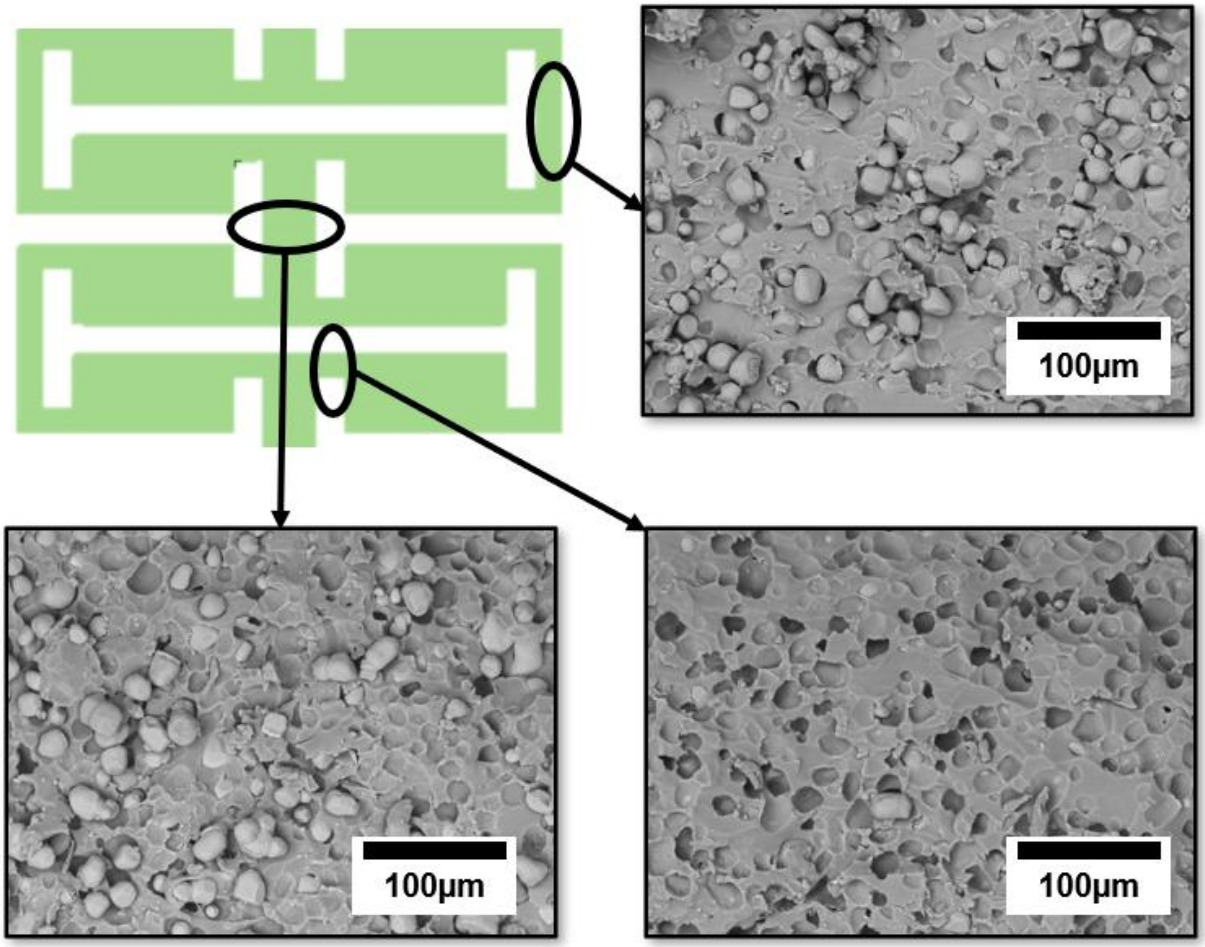

Figure S6 Cross-sectional SEM observation of Kirigami sample showing the distribution of ZnS:Cu particle on the matrix. Various cross-sections on the kirigami are also acquired, the different pictures correspond to a different section that is indicated by each respective sign and arrow.

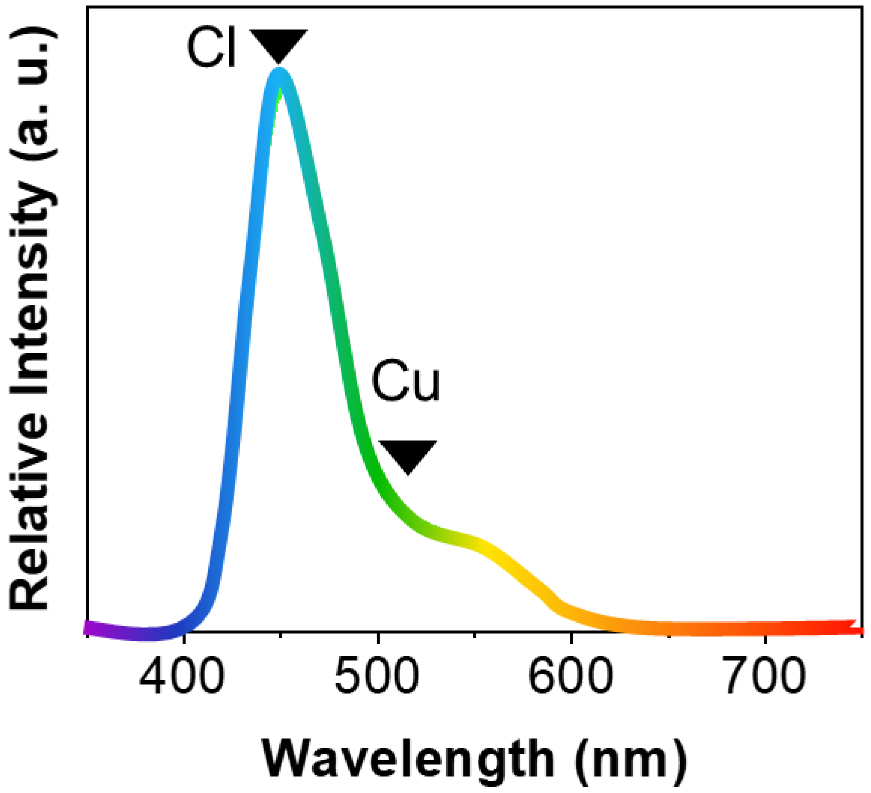

Figure S7 Photoluminescence (PL) measurement of ZnS:Cu powder, there are seems to be 2 peaks caused by the co-doping of Cu and Cl. The high peak on 450nm indicates the low Cu doping content since according to studies the peak typically skews to the right as Cu composition is increased.

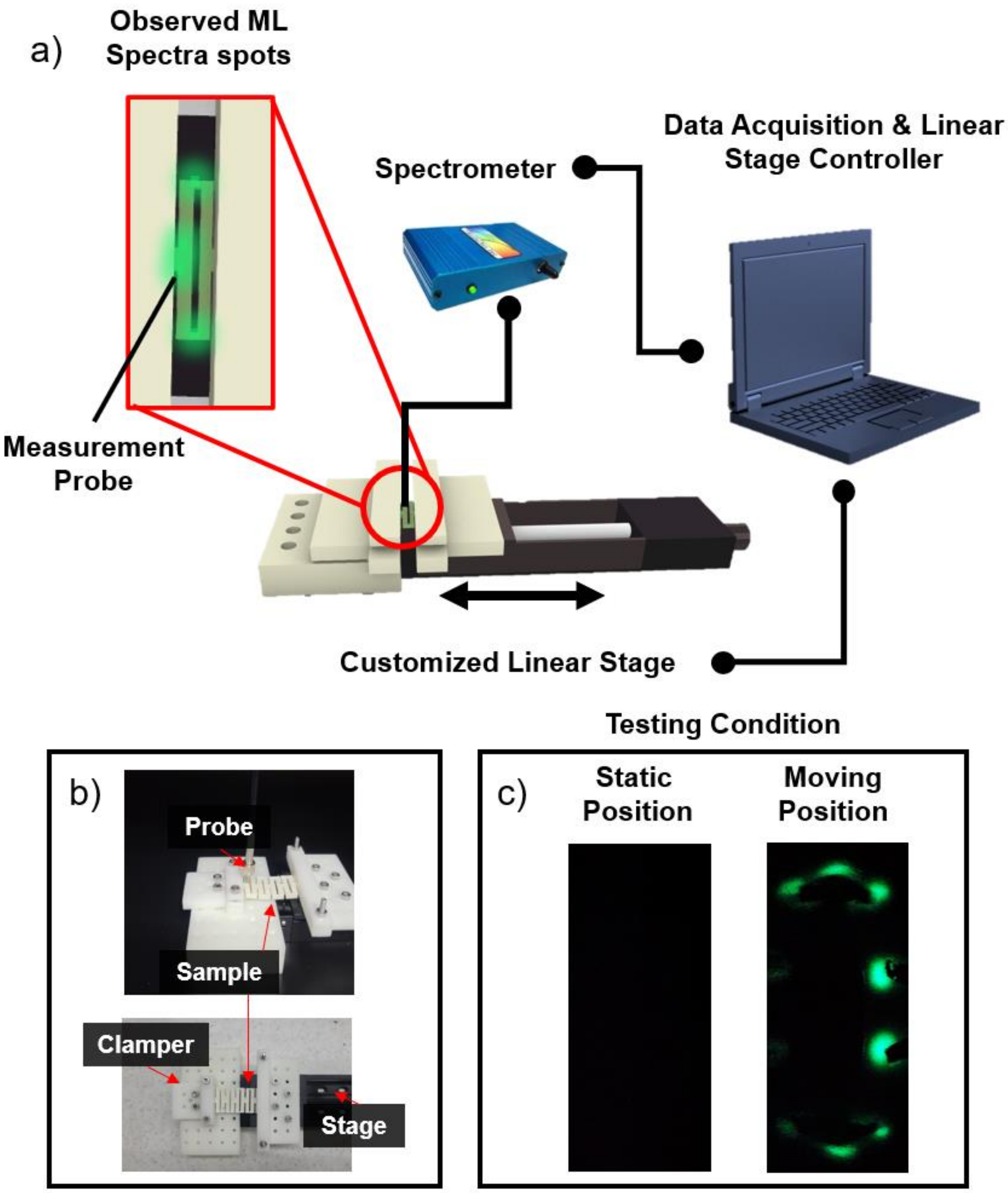

Figure S8 Mechanoluminescence (ML) characterization was done with the setup (a) comprised of a linear stage, spectrometer, and a computer used for data acquisition and stage controller. Picture (b) shows the setup placement and condition. The spectrometer probe is placed near the edge area that has high stress when applied with displacement. During the testing condition, the setup is placed in an isolated environment. In static position (c), the picture is very dark and when displacement is applied by stage (moving position) ML can be vividly observed.

	**Maximum Stress**		
**Thickness**	Kirigami Cut Shape	Kirigami Arrow Shape	Kirigami T-shape
**1 mm**	1.31 MPa	3.72 MPa	7.6 MPa
**2 mm**	0.69 MPa	1.97 MPa	3.31 MPa
**3 mm**	0.39 MPa	1.22 MPa	2.23 MPa

Table S1 The maximum stress value taken from the FEA software with the variation of different kirigami shapes and thicknesses.
